# Latent Senses

**Published:** 1872-11

**Authors:** 


### SELECTIONS.

### Latent Senses.

The shallow materialism which would confine all our knowledge to that furnished us by our five senses, meets its refutation in a close study of our physical faculties, not less decidedly than we analyze our mental powers.

No rational observer can deny the existence of certain peculiar abilities, or, if we may use the expression, latent senses, which take cognizance of facts, and convey a more or less clear impression of them to the intellect, and which are developed far more strongly in some individuals than in others. They point to qualities of matter not recognized by our weights and scales, and conditions of existence quite asunder from those with which our other senses make us familar. Misinterpreted, they make up "the night side of nature;" they become a source of superstition or pseudo-science; and sound men fight rather shy of having anything to do with them, lest they should be classed with the impositors who make capital out of them.

A simple example of what we mean is seen in certain persons of nervous temperament, who can tell the approach of an unseen and unheard individual by a kind of instinct, a sense of presence, not attributable to any sense. Others, again, less numerous, are distinctly and immediately affected by a prevailing tone of mind in an assemblage or in a companion, though no over act, or word, or look, betrays that condition. Whether this faculty is occasionally carried to such lengths as to bring about the state known as *en rapport*, we have never had an opportunity to convince ourselves, beyond the suspicion of possible deception. That there is an "atmosphere of mind," however, many facts prove. Some striking instances of "presentiments" can only thus be explained.

British naval history contains a perfectly well-authenticated instance of a commander of a vessel who altered his course, and stopped at an uninhabited island, prompted to do so by a pervading certainty that he would find shipwrecked and suffering men there. He did so, and, being the reverse of superstitious, explained it on the theory that their intense anxiety affected his mind, though at a distance, though some medium of communication similar to that which deflects certain metals, when freely suspended, toward the pole.

How far the demon of Socrates, and those other inward guides which other men of great genius have recognized, can be explained on similar hypotheses, it were premature to say. Was it an idle superstition that these men cherised? It is noticed that those who risk much and often, are most observant and heedful of these silent monitors. Is this because the slaves of chance, knowing their success depends not on careful forethought, are thereby driven to groveling superstition? Or is it not that dealing in matters where reason cannot help them, and remains silent, they are more heedful to the voice of other mentors, disregarded by most minds?

An example of this is well-known in Europe, in the person of a wealthy Maltese nobleman, the Commandatore Vincenzo Bugeja. He is often seen at the famous resorts along the Rhine where public gambling-tables are found. He plays only at rare intervals, and then only for very large stakes; but he declares that he never plays unless inwardly admonished to do so by some inward power or impulse which he cannot resist, and he always leaves off playing the instant he is warned by this same influence. He wins while obeying the impulse, but loses when he disobeys the admonition to stop. He appeared at the German tables in 1865, 1867, and 1869, and on every occasion was a heavy winner; but he distributed the greater portion of his winnings among the poor, and then left abruptly for parts unknown, and would not be heard of again for many months; indeed, after his last appearance in Homburg, in 1869, he was not seen again in Germany until about a month ago. He then made his appearance at the tables at Homburg, but remained a fortnight without staking a franc. Then, suddenly, he played with great boldness, winning twenty thousand dollars the first day, and never less than five thousand dollars on other days, "until he heard from his internal monitor," and then he started for Berlin.

But a much more satisfactory example of what we mean by latent senses is told of himself by Mr. W. Hankes Levy, F. R. G. S., in a work entitled "Blindness and the Blind," lately published in London. He says:

"Whether within a house or in the open air, whether walking or standing still, I can tell, although quite blind, when I am opposite an object, and can perceive whether it be tall or short, slender or bulky. I can also detect whether it be a solitary object or a continuous fence, whether it be a closed fence or composed of open rails, and often whether it be a wooden fence, a brick or stone wall, or a quick-set hedge. I cannot usually perceive objects if much lower than my shoulder, but sometimes very low objects can be detected. This may depend on the nature of the objects, or on some abnormal state of the atmosphere. The currents of air can have nothing to do with this powcr, as the state of the wind does not directly affect it; the sense of hearing has nothing to do with it, as when snow lies thickly on the ground objects are more distinct, although the footfall cannot be heard. I seem to perceive objects through the skin of my face, and to have the impressions immediately transmitted to the brain. The only part of my body possessing this power in my face; this I have ascertained by suitable experiments. Stopping my ears does not interfere with it, but covering my face with a thick veil destroys it altogether. None of the five senses have anything to do with the existence of this power, and the circumstances above named induce me to call this unrecognized sense by the name of 'Facial Perception.'"

He adds the following examples of the accuracy of this sense:

"When passing along a street I can distinguish shops from private houses, and even point out the doors and windows, etc., and this whether the doors be shut or open. When a window consists of one entire sheet of glass it is more difficult to discover than one composed of a number of small panes. From this it would appear that glass is a bad conductor of sensation, or at any rate of the sensation specially connected with this sense. When objects below the face are perceived, the sensation seems to come in an oblique line from the object to the upper part of the face. Whilst walking with a friend in Forest Lane, Stratford, I said, pointing to a fence which separated the road from a field, 'Those rails are not quite as high as my shoulder.' He looked at them, and said they were higher. We, however, measured, and found them about three inches lower than my shoulder. At the time of making this observation I was about four feet from the rails."

In an analagous manner, positively deaf people are aware of persons walking behind them, or of a loud noise, although of the nature of the noise they have no clear conception. The waves of sound impress them in some other way than through the nerves of audition. The subject is one that offers an extensive and attractive field for study, and will some day yield unexpectedly rich results, we do not doubt.—*Medical and Surgical Reporter.*

